# Taxonomic Positions and Secondary Metabolite-Biosynthetic Gene Clusters of Akazaoxime- and Levantilide-Producers

**DOI:** 10.3390/life13020542

**Published:** 2023-02-15

**Authors:** Hisayuki Komaki, Tomohiko Tamura, Yasuhiro Igarashi

**Affiliations:** 1 Biological Resource Center, National Institute of Technology and Evaluation (NBRC), Chiba 292-0818, Japan; 2 Biotechnology Research Center and Department of Biotechnology, Toyama Prefectural University, Toyama 939-0398, Japan

**Keywords:** akazaoxime, A-76356, biosynthesis, classification, levantilide, *Micromonospora*, non-ribosomal peptide, polyketide

## Abstract

*Micromonospora* sp. AKA109 is a producer of akazaoxime and A-76356, whereas *Micromonospora* sp. AKA38 is that of levantilide C. We aimed to clarify their taxonomic positions and identify biosynthetic gene clusters (BGCs) of these compounds. In 16S rRNA gene and DNA gyrase subunit B gene (*gyrB*) sequence analyses, strains AKA109 and AKA38 were the most closely related to *Micromonospora humidisoli* MMS20-R2-29^T^ and *Micromonospora schwarzwaldensis* HKI0641^T^, respectively. Although *Micromonospora* sp. AKA109 was identified as *M. humidisoli* by the *gyrB* sequence similarity and DNA–DNA relatedness based on whole genome sequences, *Micromonospora* sp. AKA38 was classified to a new genomospecies. *M. humidisoli* AKA109 harbored six type-I polyketide synthase (PKS), one type-II PKS, one type-III PKS, three non-ribosomal peptide synthetase (NRPS) and three hybrid PKS/NRPS gene clusters, among which the BGC of akazaoxime and A-76356 was identified. These gene clusters are conserved in *M. humidisoli* MMS20-R2-29^T^*. Micromonospora* sp. AKA38 harbored two type-I PKS, one of which was responsible for levantilide C, one type-II PKS, one type-III PKS, two NRPS and five hybrid PKS/NRPS gene clusters. We predicted products derived from these gene clusters through bioinformatic analyses. Consequently, these two strains are revealed to be promising sources for diverse non-ribosomal peptide and polyketide compounds.

## 1. Introduction

**Figure 1:**
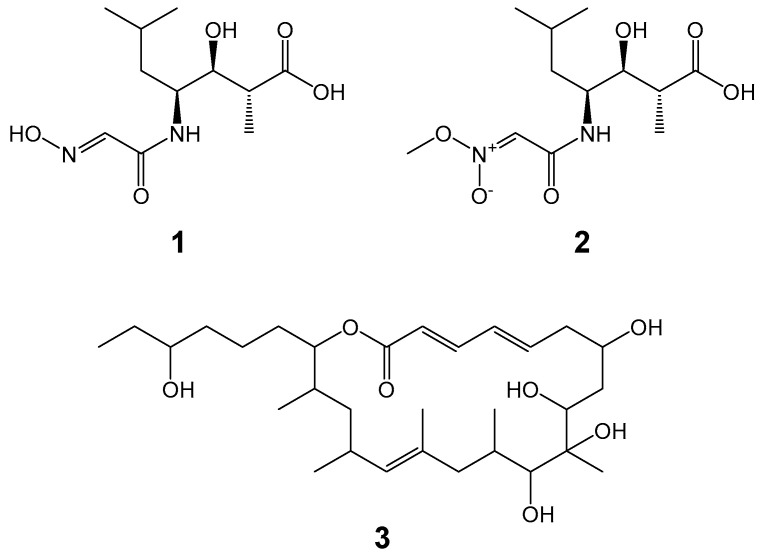
Chemical structures of akazaoxime (**1**), A-76356 (**2**) and levantilide C (**3**).

Actinomycetes are Gram stain-positive and filamentous bacteria with high G + C contents in genomic DNAs. They are well known as a promising source for pharmacologically useful bioactive substances with diverse chemistries, from which many pharmaceuticals were developed and are clinically used [1]. The genus *Streptomyces* is the representative of actinomycetes, and its main habitat is soil. However, soil environments are extensively searched for novel actinomycetes, and consequently, it is getting harder to isolate novel actinomycetal strains from the same environments. In contrast, marine environments are attracting attention as rich sources of underexplored actinomycetes. Indeed, we have discovered new and diverse bioactive secondary metabolites from marine actinomycetes [2,3,4,5,6,7,8,9]. *Micromonospora* strains are frequently isolated from marine environments. Many bioactive substances are reported from this genus [10,11]. We previously isolated *Micromonospora* sp. AKA109 and *Micromonospora* sp. AKA38 from deep sea water. From *Micromonospora* sp. AKA109, a new compound named akazaoxime (**1**, Figure 1) was discovered, along with a known compound, A-76356 (**2**, Figure 1). Akazaoxime and A-76356 are enteromycin-class antibiotics. Incorporation experiments of labelled precursors suggested these two compounds are biosynthesized from glycine, leucin and propionate. Akazaoxime exhibits antibacterial activity to Gram-positive *Kocuria rhizophila,* whereas A-76356 is active against filamentous fungi such as the plant pathogen *Glomerella cingulata* [12]. *Micromonospora* sp. AKA38 produces levantilide C (**3**, Figure 1), which is a 20-membered macrolide and exhibits antiproliferative activities against several tumor cell lines [13]. Biosynthetic gene clusters (BGCs) of these compounds have not been identified yet, although identification of BGCs plays an important role in developments in combinatorial biosynthesis and synthetic biology.

Polyketides such as macrolide backbones are biosynthesized by the assemblage of acyl-CoAs as building blocks. The assembly is catalyzed by polyketide synthases (PKSs). PKSs are classified by three types. Type-I PKSs are large modular enzymes composed of multiple catalytic domains. Polyketide chains are synthesized according to the co-linearity rule of assembly lines. Such a mechanism shows similarity to that in the biosynthesis of non-ribosomal peptides by non-ribosomal peptide synthetases (NRPSs), which is based on assembly of amino acids as building blocks. NRPSs as well as type-I PKSs are large and modular enzymes with multiple catalytic domains, and they accord to the co-linearity rule [14,15]. Polyketide chains for macrolide compounds are synthesized by type-I PKSs. Backbones synthesized by type-I PKSs and/or NRPSs can be predicted from their domain organizations by bioinformatic analysis [14,15]. In contrast, type-II PKSs are composed of three monofunctional enzymes, ketosynthase α (KSα), KSβ (chain length factor), and acyl carrier protein (ACP). Differently from type-I PKSs, these three enzymes iteratively catalyze multiple chain elongation steps. The main products of type-II PKSs are aromatic compounds [16]. Type-III PKSs are not multimodular or composed of abovementioned three enzymes, but stand alone with a KS domain and iteratively catalyze the assembly of the acyl-CoA unit [17]. Genome analyses revealed that half to three quarters of the secondary metabolite-BGCs in each actinomycetal genome are associated with PKSs or NRPSs. This suggests that polyketides, non-ribosomal peptides, and their hybrid compounds, which are derived from hybrid PKS/NRPS gene clusters, are main secondary metabolites in actinomycetes [18].

In the present study, we classified *Micromonospora* sp. AKA109 and *Micromonospora* sp. AKA38 at species level. Next, we identified BGCs for akazaoxime/A-76356 and levantilide C through analysis of PKS and NRPS gene clusters in their genomes. The analysis revealed the potential of the two strains to act as producers of diverse polyketide- and nonribosomal peptide-compounds. These results are useful to elucidate potential products of each strain.

## 2. Materials and Methods

*Micromonospora* strains AKA109 and AKA38 were isolated from deep sea water collected in Shizuoka, Japan, maintained as TP-A0907 and TP-A0908, respectively, in Toyama Prefectural University, and have been deposited to and are available from the NBRC culture collection as NBRC 113680 and NBRC 113681, respectively. The 16S rRNA genes were amplified by PCR using 9F and 1541R primers. The amplicons were sequenced by the method described in our previous report [19]. Type strains showing high 16S rRNA gene sequence similarities to AKA109 and AKA38 were searched using the EzBioCloud web server [20]. Phylogenetic trees based on 16S rRNA gene and DNA gyrase subunit B gene (*gyrB*) sequences were reconstructed by the neighbor-joining method using ClustalX 2.1. Whole genomes were sequenced using PacBio, as reported [21]. Draft genome sequences of strains AKA109 and AKA38 were deposited to DDBJ under the accession numbers of BNEH01000001–BNEH01000007 and BNEI01000001–BNEI01000011, respectively. A phylogenomic tree was reconstructed using the TYSG server [22]. DNA–DNA relatedness was calculated by digital DNA–DNA hybridization (DDH) using the Genome-to-Genome Distance Calculator 2.1 (GGDC) [23], and DDH estimates by the Formula 2 were employed. PKS and NRPS gene clusters in the whole genome were searched, and their domains were determined using antiSMASH [24]. The products were predicted by reviewing module numbers and domain organizations in PKSs and NRPSs, the substrates of acyltransferase (AT) and adenylation (A) domains, and orthologs searched by BLAST, in addition to results of ClusterBlast in antiSMASH.

## 3. Results

### 3.1. Classification of Micromonospora Strains AKA109 and AKA38

**Figure 2:**
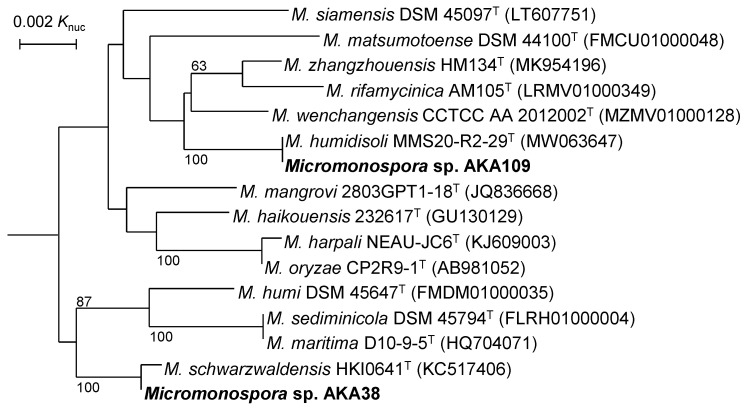
Phylogenetic tree based on 16S rRNA gene sequences. Type strains of species showing sequence simiralities of >99.0% to *Micromonospora* sp. AKA109 and/or *Micromonospora* AKA38 are included in this tree. Numbers on the branches are the confidence limits estimated by bootstrap analysis with 1000 replicates, and values above 50% are indicated at branching points. *Phytohabitans suffuscus* K07-0523^T^ (AB490769) was used as an outgroup (not shown).

In the 16S rRNA gene sequence analysis, *Micromonospora* sp. AKA109 showed 100% similarity to *Micromonospora humidisoli* MMS20-R2-29^T^, whereas *Micromonospora* sp. AKA38 showed 99.9% similarity to *Micromonospora schwarzwaldensis* HKI0641^T^ as the closest. In the phylogenetic tree shown in Figure 2, strain AKA109 formed an independent clade with *M. humidisoli* MMS20-R2-29^T^, whereas strain AKA38 did that with *M. schwarzwaldensis* HKI0641^T^.

**Figure 3:**
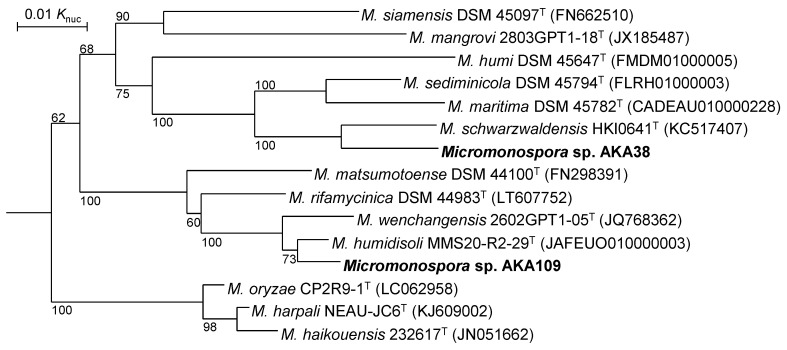
Phylogenetic tree based on *gyrB* sequences. Type strains of species shown in Figure 2 are included in this tree. Numbers on the branches are the confidence limits estimated by bootstrap analysis with 1000 replicates, and values above 50% are indicated at branching points. *P. suffuscus* NBRC 105367^T^ (AP022871) was used as an outgroup (not shown).

We next reconstructed a phylogenetic tree based on *gyrB* sequences, as shown in Figure 3, since *gyrB* sequences are recognized to be more suitable than 16S rRNA gene sequences for phylogenetic classification and identification [25]. In this tree, *M. humidisoli* and *M. schwarzwaldensis* were also phylogenetically the closest species of strains AKA109 and AKA38, respectively. The *gyrB* sequence similarity between *Micromonospora* sp. AKA109 and *M. humidisoli* MMS20-R2-29^T^ was 99.0%. Since 98.5% in *gyrB* sequence similarity is recognized to correspond to 70% in DNA–DNA relatedness [25,26], *Micromonospora* sp. AKA109 is likely *M. humidisoli*. In contrast, the *gyrB* sequence similarity between *Micromonospora* sp. AKA38 and *M. schwarzwaldensis* HKI0641^T^ was 97.4%, which is much below than 98.5%; therefore, *Micromonospora* sp. AKA38 is considered an independent new genomospecies.

**Figure 4:**
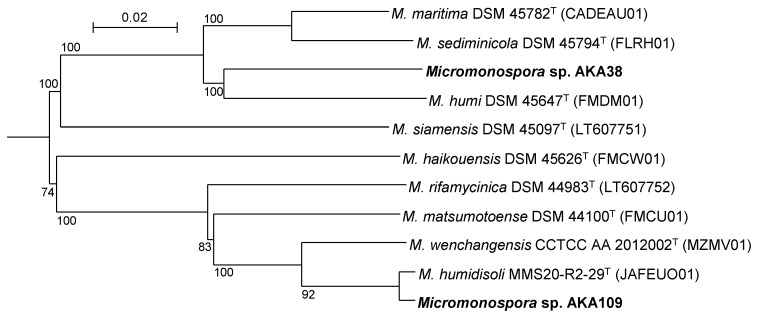
Phylogenomic tree reconstituted using the TYGS server. *P. suffuscus* NBRC 105367^T^ (AP022871) was used as an outgroup (not shown) to show the root. The numbers in parentheses are accession numbers of WGS Projects or whole genome sequences in GenBank. Type strains of species shown in Figure 2 whose whole genome sequences are published are included in this tree.

Additionally, a phylogenomic tree was reconstructed with type strains whose whole genome sequences are published (Figure 4). The phylogenetic relationships well correlated to those in phylogenetic trees of Figure 1 and Figure 2. DNA–DNA relatedness, estimated by digital DDH, between *Micromonospora* sp. AKA109 and *M. humidisoli* MMS20-R2-29^T^ was 93.5%. As this value is much higher than 70%, which is the established cut-off for species delineation [27,28,29], strain AKA109 was identified to be *M. humidisoli.* In contrast, DNA–DNA relatedness between *Micromonospora* sp. AKA38 and the other strains shown in Figure 4 were less than 41.4%. This result also shows *Micromonospora* sp. AKA38 to be an independent genomospecies.

### 3.2. PKS and NRPS Gene Clusters in the Whole Genome of M. humidisoli AKA109

Six type-I PKS, one type-II PKS, one type-III PKS, three NRPS and three hybrid PKS/NRPS gene clusters were encoded in the genome of *Micromonospora* sp. AKA109. Type-I PKS gene cluster 1 (*t1pks-1*) encoded three PKSs, whose domain organization was almost identical to those (KS AT_m_ ACP KS AT_m_ DH KR ACP KS AT_m_ DH KR ACP, KS AT_m_ DH KR ACP KS AT_m_ DH KR ACP, KS AT_m_/_mm/em_ DH KR ACP TD) of camporidine-, argimycin- and streptazone-BGCs [30,31,32]. However, *t1pks-1* lacked the KR domain (underlined in the previous brackets) present in CamD, ArpII and StzC. Although the substrate of the last AT domain in *t1pks-1* was methylmalonyl-CoA, those in ArpIII and StzB are malonyl-CoA. Thus, product(s) of *t1pks-1* may resemble camporidine, argimycin or streptazone, but will be different from these. PKSs encoded in *t1pks-2, t1pks-3* and *t1pks-4* did not show high sequence similarities to PKSs whose products have been identified. Thus, the products of these PKS gene clusters were not predicted. The domain organization, KS/AT/KR/DH, of the PKS encoded by TPA0907_18690 in *t1pks-3* is well known as that of iterative PKSs for enediyne syntheses. Hence, the products of *t1pks-3* may include an enediyne moiety. *T1pks-5* encoded five PKSs. These PKSs showed high similarities to those in the marinolactam-BGC (*mrl*) [33]. Their domain organization was identical to that of *mrl* except for the presence of a DH domain in the first module of MrlB, which is absent in that of TPA0907_35890. Therefore, we annotated this cluster to be responsible for a marinolactum congener. As genes in *t1pks-6* showed high similarities to those in the amycomicin-BGC, the product was predicted to be amycomicin. Products of type-II PKS gene cluster 1 (*t2pks-1*) were predicted to be an aromatic compound. Type-III PKS gene cluster 1 (*t3pks-1*) showed similarity to *agq,* which is the BGC of alkyl-*O*-dihydrogeranyl-methoxyhydroquinone [34]. Three NRPS gene clusters (*nrps-1*, *nrps-2,* and *nrps-3*) did not show high similarities to those whose products are elucidated, suggesting that they are orphan gene clusters. Although the product of *nrps-2* was unpredictable because its NRPS was not multimodular, those of *nrps-1* and *nrps-3* were predicted as dipeptide and tetrapeptide, respectively, as shown in Table 1. Hybrid PKS/NRPS gene clusters 1 and 2 (*pks/nrps-1* and *pks/nrps-2*) were orphan. The domain organization of *pks/nrps-1* was unusual, because thioesterase (TE) domain is not present at the terminal, but as the first domain. Hence, it is doubtful that the cluster works to synthesize hybrid polyketide/non-ribosomal peptide compounds. The product derived from *pks/nrps-2* was predicted to be a hybrid polyketide/non-ribosomal peptide compound including Asn and Ser residues.

**Figure 5:**
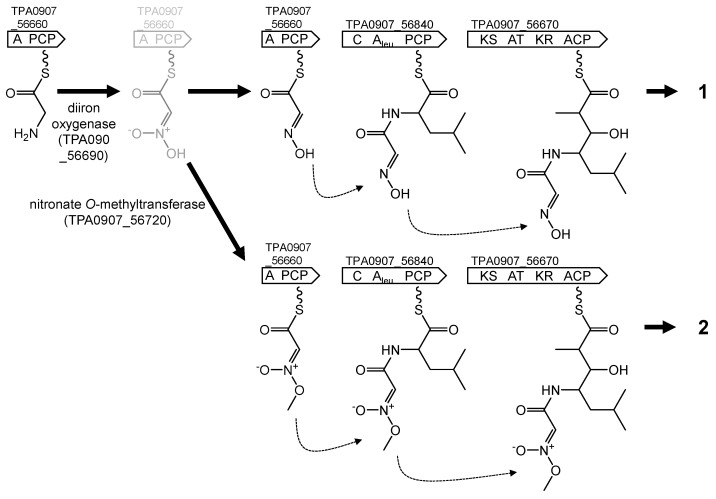
Putative biosynthetic pathways for akazaoxime (**1**) and A-76356 (**2**). An intermediate converted by the diiron oxygenase is shown in gray.

We considered *pks/nrps-3* to be the BGC for akazaoxime and A-76356, according to its domain organization and the biosynthetic pathway revealed by incorporation of labeled precursors [12]. These two compounds have been reported to be synthesized from glycine, leucine, and propionate. Similarly, *pks/nrps-3* encodes two NRPS and one PKS, which incorporate two amino acids and one acyl-CoA, respectively, to the product. One of the amino acids was predicted to be leucine, although the other was bioinformatically not. Presence of a KR domain in the PKS well accounts for hydration of the keto group derived from carboxyl group of leucine. The cluster encoded a diiron oxygenase and a nitronate *O*-methyltransferase, which are essential to form aldoxime functionality and an *O*-methyl nitronic acid moiety [35]. We predicted the biosynthetic pathway of akazaoxime and A-76356, as shown Figure 5. A glycine molecule is loaded on the NRPS encoded by TPA0907_56660. Its amino group is converted to an aldoxime functionality through an intermediate by the diiron oxygenase, as reported in the biosynthesis of althiomycin [35,36]. If the methyltransferase encoded by TPA0907_56720 acts the intermediate, the amino group is converted to *O*-methyl nitronic acid moiety, as reported in the biosynthesis of enteromycin carboxamide [35]. To the modified glycine molecules, leucine and methylmalonyl-CoA are bound by the other NRPS (TPA0907_56840) and the PKS (TPA0907_56670). Finally, the chains are released from the PKS to yield akazaoxime (**1**) and A-76356 (**2**), respectively.

### 3.3. PKS and NRPS Gene Clusters in the Whole Genome of Micromonospora sp. AKA38

*Micromonospora* sp. AKA38 harbored two type-I PKS, one type-II PKS, one type-III PKS, two NRPS and five hybrid PKS/NRPS gene clusters in its genome, as listed in Table 2.

**Figure 6:**
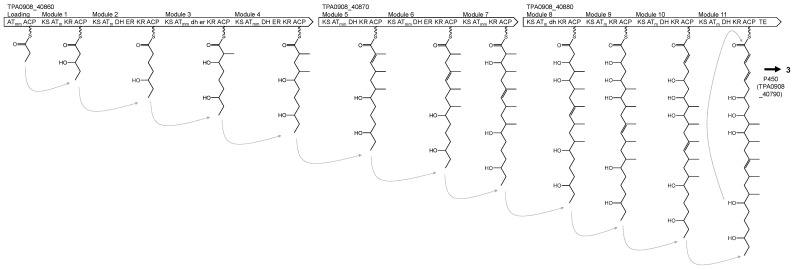
Proposed biosynthetic pathway of levantilide C (**3**). Abbreviations of domains are the same as those in Table 1. dh, inactive DH; er, inactive ER.

We annotated *t1pks-7* as the BGC of levantilide C, according to its domain organization and the chemical structure. The cluster encoded three PKSs including a loading module and eleven modules to incorporate acyl-CoAs in the polyketide chain, as shown in Figure 6. The chemical structure predicted by the domain organization well matched to that of levantilide C. DH and ER domains in module 3 and the DH domain in module 8 would be inactive considering the actual chemical structure of levantilide C. A hydroxyl group is present at C-10 in levantilide C, and it does not form by polyketide biosynthesis. Because a cytochrome P450 is encoded near the PKSs in the gene cluster as TPA0908_40790, the hydroxyl group is likely introduced by the cytochrome P450.

*T1pks-8* is a large type-I PKS gene cluster encoding 13 PKSs, which form 33 modules. The product was predicted to be quinolidomicin based on the domain organization and similarities to quinolidomicin’s PKSs (QmnA1 to QmnA13) [37]. The gene cluster is widely distributed in the genus *Micromonospora* [38]. The product of *t2pks-2* could not be predicted because the type-II PKSs did not show high sequence similarities to enzymes for the reported compounds. In most type-II PKS gene clusters, an ACP is encoded downstream of KSβ (CLF), but the ACP of *t2pks-2* is upstream of KSα and includes a cyclase domain. Two gene clusters, *t3pks-1* and *pks/nrps-2*, asterisked in the tables, were orthologs of those present in *M. humidisoli* AKA109. The other gene clusters, such as *nrps-4, nrps-5, pks/nrps-4, pks/nrps-5, pks/nrps-6* and *pks/nrps-7,* were orphan, and their products were predicted as shown in Table 2. In *pks/nrps-7*, two type-I PKSs whose domain organizations are KS-AT-KR-DH and KS-AT-ACP, respectively and one type-III PKS were encoded in addition to NRPSs. The domain pair, KR-DH, observed in one of the type-I PKSs is known to be specific for PksE. Therefore, the product of *pks/nrps-7* will include an enediyne moiety [39].

### 3.4. Specificity of the PKS and NRPS Gene Clusters in Each Strain

We conducted a BLAST search to investigate whether the gene clusters identified in this study are specific in each strain or present in the other strains. All the PKSs and NRPSs of *M. humidisoli* AKA109 were also present in *M. humidisoli* MMS20-R2-29^T^ (Table 3). As the TPA0907_16820 homolog in *M. humidisoli* MMS20-R2-29^T^ is not well sequenced, it was not hit in the search. Although a homolog of TPA0907_20190 was also present in *M. humidisoli* MMS20-R2-29^T^, it is not described in the table because its sequence identity/similarity were lower (99/98 in%) than those of *Micromonospora* sp. RL09-050-HVF-A.

Among eleven gene clusters of *Micromonospora* sp. AKA38, seven (*t1pks-8, t2pks-2, t3pks-1, pks/nrps-2, pks/nrps-4, pks/nrps-5* and *pks/nrps-7*) were present in other strains with high sequence identity/similarity, although TPA0908_54560, TPA0908_54550 and TPA0908_54470 in *pks/nrps-7* were not observed, suggesting *pks/nrps-7* orthologs in other strains may be partial or not completely sequenced. Except for *pks/nrps-4*, the closest genes were present in *Micromonospora* sp. RP3T and their identity/similarity values were quite high. In contrast, four gene clusters, *t1pks-7, nrps-4, nrps-5* and *pks/nrps-6,* were not present in other strains because their BLAST top hits showed low identity/similarity values. This suggests that they are novel and specific to strain AKA38.

## 4. Discussion

Many strains found as producers of new bioactive substances have not been classified yet at species level. Consequently, relationships between products and taxonomic positions of the producer are not well understood. In this study, we classified *Micromonospora* sp. AKA109, a producer of akazaoxime and A-76356, to *M. humidisoli* [40]. In contrast, *Micromonospora* sp. AKA38, a producer of levantilide C, was revealed to be a novel genomospecies. If *Micromonospora* sp. AKA38 is characterized in detail [41], it can be proposed as a new *Micromonospora* species because it was not classified to known species. *M. humidisoli* is very recently proposed, and its type strain, MMS20-R2-29^T^, was isolated from riverside soil. It is explained that its growth occurs in the presence of 0–2% NaCl, with optimal growth at 0% NaCl [40]. In contrast, strain AKA109 was isolated from deep sea water with a higher salt concentration. To the best of our knowledge, this is the first report on marine-derived *M. humidisoli*.

Recently, genome mining has often been used when searching for new compounds. However, if researchers find an unknown BGC that appears novel by genome mining, it may be a BGC for known compounds, because many BGCs of known compounds have not been identified, and consequently, they are considered BGCs for new compounds. Thus, BGCs of known compounds need to be identified for more effective genome mining if the BGCs have not been unidentified. We here identified the BGC of akazaoxime and A-76356, and that of levantilide C from *Micromonospora* sp. AKA109 and *Micromonospora* sp. AKA38, respectively. This is the first report on the BGCs and biosynthetic pathways of these compounds.

*Micromonospora* sp. AKA109, classified to *M. humidisoli*, harbored fourteen PKS and NRPS gene clusters, all of which are also present in *M. humidisoli* MMS20-R2-29^T^. This well supports our idea that members of the same species possess similar sets of PKS and NRPS gene clusters [42,43,44]. *Micromonospora* sp. AKA38, classified as a new genomospecies, harbored eleven PKS and NRPS gene clusters. Although seven of them were present in other strains, such as *Micromonospora* sp. RP3T and *Micromonospora* sp. WMMA2032, the remaining four are not found in any other strains. If a strain is taxonomically novel at the species level, it may possess new PKS and/or NRPS gene clusters.

Although PKS and NRPS gene clusters found from our two strains include BGCs of known compounds such as amycomicin, alkyl-*O*-dihydrogeranyl-methoxyhydroquinone and quinolidomicin, and congeners of known compounds, they include many orphan and unknown clusters. Their products were predicted to be novel at present. Thus, these two strains are expected to produce new and diverse polyketide and non-ribosomal peptide compounds.

Except for PKS and NRPS gene clusters, eleven putative secondary metabolite-biosynthetic gene clusters are present in each genome of *M. humidisoli* AKA109 and *Micromonospora* sp. AKA38 (Tables S1 and S2). The products, except for SapB, desferrioxamine, *N*-acetylglutaminylglutamine amide (NAGGN) and class II lanthipeptides of *Micromonospora* sp. AKA38, could not be predicted because there is less information on these types of gene clusters. SapB, desferrioxamine, NAGGN, three terpene, and one hybrid oligosaccharide/terpene gene cluster are conserved in the two strains. SapB, desferrioxamine and NAGGN are known as common secondary metabolites in actinomycetes. The numbers of gene clusters shown in Tables S1 and S2 did not exceed those of the PKS and NRPS gene clusters (Table 1 and Table 2). This supports the assertion that polyketides and non-ribosomal peptides are major and diverse secondary metabolites, as previously reported [18].

## 5. Conclusions

We sequenced whole genomes of an akazaoxime- and A-76356-producer, *Micromonospora* sp. AKA109, and a levantilide C-producer, *Micromonospora* sp. AKA38. *Micromonospora* sp. AKA109 was identified as *M. humidisoli,* whereas *Micromonospora* sp. AKA38 was revealed to be a new genomospecies. Akazaoxime- and A-76356-BGC and levantilide C-one were identified from whole genome sequences of these two strains, respectively. *M. humidisoli* AKA109 harbored fourteen PKS and NRPS gene clusters, all of which were conserved in the type strain of *M. humidisoli*. *Micromonospora* sp. AKA38 harbored eleven PKS and NRPS gene clusters. Our bioinformatic analysis suggested their potential to synthesis diverse non-ribosomal peptides and polyketides.

## Figures and Tables

**Table 1:** PKS and NRPS gene clusters in the whole genome of *M. humidisoli* AKA109.

Cluster	Locus Tag (TPA0907)	Domain Organization	Product Predicted
*t1pks-1*	_14850 _14840 _14830	KS AT_m_ ACP KS AT_m_ DH KR ACP KS AT_m_ DH KR ACP KS AT_m_ DH KR ACP KS AT_m_ DH ACP KS AT_mm_ DH KR ACP TD	New analog(s) of camporidine, argimycin, streptazone
*t1pks-2*	_16830 _16820 _16810	KS AT_m_ ACP ACP ACP KR KS AT_mm_ ACP	Unknown
*t1pks-3*	_18400 _18690	KS AT DH KR ACP KS AT_m_ KR DH	Compound with an enediyne moiety
*t1pks-4*	_47680	KS AT_m_ DH ER KR ACP	Unknown
*t1pks-5* (*mrl*)	_35900 _35890 _35880 _35870 _35750	KS AT_m_ DH KR ACP KS AT_m_ KR ACP KS AT_m_ KR ACP KS AT_mm_ KR ACP KS AT_m_ DH KR ACP KS AT_m_ DH KR ACP KS AT_m_ DH KR ACP TE ACP KS AT_m_ DH KR ACP KS AT_mm_ DH KR ACP KS AT_mm_ DH KR ACP KS AT_m_ DH KR ACP	Marinolactam congener
*t1pks-6*	_29310	KS AT_m_ DH ER KR ACP	Amycomicin
*t2pks-1*	_20160 _20170 _20190	KSα KSβ (CLF) ACP	Aromatic polyketide
*t3pks-1* * (*aqq*)	_59200	KS	Alkyl-*O*-dihydrogeranyl-methoxyhydroquinone
*nrps-1*	_47220 _47230 _47240	C A_phe_ PCP C A PCP C	Phe-x
*nrps-2*	_47680	A PCP C	Unknown
*nrps-3*	_56920 _56930 _56940 _56970 ^C^	C A PCP C A_cys_ PCP A PCP C C A_glu_ PCP E C	Tetrapeptide including Cys and Glu
*pks/nrps-1*	_15480	TE A PCP KS AT_m_ KR ACP	Unknown
*pks/nrps-2* *	_28040 _28030 _28010 _28000 _27970 _27960	A PCP KS TE A PCP C PCP KS AT_m_ KR DH ACP C A_asn_ PCP C A_ser_ PCP TE	x-x-y-mal-Asn-Ser
*pks/nrps-3*	_56660 _56670 _56710 _56840 ^C^	A PCP KS AT_m_ KR ACP ACP C A_leu_ PCP	Akazaoxime and A-76356

^C^, encoded in the complementary strand; *, conserved between strains AKA109 and AKA38; A, adenylation domain; ACP, acyl carrier protein; AT, acyltransferase domain; AT_m_, AT for malonyl-CoA, AT_mm_, AT for methylmalonyl-CoA; AT_em/mx_, AT for ethylmalonyl-CoA or methoxymalonyl CoA; C, condensation domain; CLF, chain length factor; CoL, CoA ligase domain; DH, dehydratase domain; Cyc, cyclase domain; E, epimerization domain; ER, enoylreductase domain; KR, ketoreductase domain; KS, ketosynthase domain; mal, residue derived from malonyl-CoA; MT, methyltransferase domain; *nrps*, PCP, peptidyl carrier protein; *nrps*, NRPS gene; *pks/nrps*, hybrid PKS/NRPS gene; pk, residue derived from a single module of type-I PKS; TD, termination domain; TE, thioesterase domain, *t1pks*, type-I PKS gene; *t2pks*, type-II PKS gene; *t3pks*, type-III PKS gene; x, unidentified amino acid residue; y, unknown unit by lack of A domain in the module. Amino acids incorporated by A domains are indicated as 3-letter abbreviations in subscript just after A.

**Table 2:** PKS and NRPS gene clusters in the whole genome of *Micromonospora* sp. AKA38.

Gene Cluster	Locus Tag (TPA0908)	Domain Organization	Product Predicted
*t1pks-7*	_40860 _40870 _40880	AT_mm_ ACP KS AT_m_ KR ACP KS AT_m_ DH ER KR ACP KS AT_mm_ DH ER KR ACP KS AT_mm_ DH ER KR ACP KS AT_mm_ DH KR ACP KS AT_mm_ DH ER KR ACP KS AT_mm_ KR ACP KS AT_m_ DH KR ACP KS AT_m_ KR ACP KS AT_m_ DH KR ACP KS AT_m_ DH KR ACP TE	Levantilide C
*t1pks-8* (*qmn*)	_45370 _45410 _45420 _45440 _45450 _45460 _45470 _45480 _45490 _45500 _45510 _45520 _45530	CoL ACP KS AT_m_ DH KR ACP KS AT_mm_ DH ER KR ACP KS AT_m_ DH KR ACP KS AT_m_ KR ACP KS AT_m_ KR ACP KS AT_m_ KR ACP KS AT_m_ DH ER KR ACP KS AT_mm_ DH ER KR ACP KS AT_m_ DH ER KR ACP KS AT_m_ KR ACP KS AT KR ACP KS AT_m_ DH KR ACP KS AT_m_ DH KR ACP KS AT_m_ DH KR ACP KS AT_m_ KR ACP KS AT_m_ KR ACP KS AT_m_ KR ACP KS AT_mm_ DH KR ACP KS AT_m_ DH KR ACP KS AT_mm_ KR ACP KS AT_m_ KR ACP KS AT_mm_ KR ACP KS AT_mm_ DH ER KR ACP KS AT_m_ KR ACP KS AT_mm_ KR ACP KS AT_mm_ KR ACP KS AT_m_ DH KR ACP KS AT_mm_ DH KR ACP KS AT_mm_ KR ACP KS AT_mm_ KR ACP KS AT_m_ KR ACP KS AT_m_ KR ACP TE	Quinolidomicin
*t2pks-2*	_49930 _49910 _49900	ACP Cyc KSα KSβ (CLF)	Unknown
*t3pks-1* * (*aqq*)	_06420	KS	Alkyl-*O*-dihydrogeranyl-methoxyhydroquinone
*nrps-4*	_34180 _34160 _34150 _34130 _34100	A_thr_ MT PCP C A_pro_ PCP C PCP C PCP TE TE A_val_ PCP A C A_thr_ PCP C A_leu_ PCP C A_pro_ PCP C A_leu_ PCP C	Val-Thr-Leu-Pro-Leu-mThr-Pro-y-y
*nrps-5*	_34870 ^C^ _34920 _35060 _35080	C A PCP C A PCP C A_asn_ PCP TE A PCP C A_asn_ PCP C A PCP C A_thr_ PCP C A_asn_ PCP C A PCP C A PCP C A_thr_ PCP C A PCP TE	x-Asn-x-Thr-Asn-x-x-Thr-x-x-x-Asn
*pks/nrps-2* *	_42740 _42750 _42770 _42780 _42810 _42820	A PCP KS TE A PCP C PCP KS AT_m_ KR DH ACP C A_asn_ PCP C A_ser_ PCP TE	x-x-y-mal-Asn-Ser
*pks/nrps-4*	_08330 _08340 _08370	C A_asn_ PCP KS AT_m_ ACP C A PCP A_ala_ PCP C A_glu_ PCP C PCP	Asn-mal-x-Ala-Glu-y
*pks/nrps-5*	_34600 _34620 _34650 _34660 _34670 _34690 _34690 _34730	TE A PCP A PCP KS A PCP C PCP KS AT_m_ KR ACP C A_ser_ PCP PCP	x-x-x-y-mal-Ser
*pks/nrps-6*	_35130 _35200 _35210 _35230 ^C^ _35250 ^C^	A_thr_ PCP A PCP C A_asn_ PCP ACP KS AT DH KR ACP C A PCP C	x-Thr-x-Asn-pk
*pks/nrps-7*	_54560 ^C^ _54550 ^C^ _54470 ^C^ _54430 ^C^ _54260 _54200 _54120 _54020 ^C^ _54000 _53990 _53970	C A PCP PCP C A A_ala_ PCP C A_val_ PCP KS (type III PKS) KS AT_m_ KR DH PCP TE C A_val_ PCP KS AT_m_ ACP A_ser_	Ala-Val-enediyne-Val-mal-Ser-x-x with an aromatic moiety

Footnotes are the same as those of Table 1.

**Table 3:** The closest homolog or ortholog of PKSs and NRPSs encoded by the gene clusters of *M. humidisoli* AKA109 and *Micromonospora* sp. AKA38.

Cluster	Locus Tag (TPA090)	BLAST Top Hit		
		I/S (%) ^1^	Locus Tag or Gene (Accession No.)	Origin
*t1pks-1*	7_14850 7_14840 7_14830	99/99 99/99 99/99	JQN84_27510 JQN84_31080 JQN84_29090	*M. humidisoli* MMS20-R2-29^T^ *M. humidisoli* MMS20-R2-29^T^ *M. humidisoli* MMS20-R2-29^T^
*t1pks-2*	7_16830 7_16820 7_16810	90/92 99/99 100/100	J7462_RS07410 JQN84_30180 JQN84_30185	*Micromonospora* sp. RL09-050-HVF-A *M. humidisoli* MMS20-R2-29^T^ *M. humidisoli* MMS20-R2-29^T^
*t1pks-3*	7_18400 7_18690	99/100 99/100	JQN84_22230 JQN84_22370	*M. humidisoli* MMS20-R2-29^T^ *M. humidisoli* MMS20-R2-29^T^
*t1pks-4*	7_47680	99/99	JQN84_24840	*M. humidisoli* MMS20-R2-29^T^
*t1pks-5* (*mrl*)	7_35900 7_35890 7_35880 7_35870 7_35750	99/99 99/99 99/99 99/99 99/99	JQN84_05260 JQN84_05265 JQN84_05270 JQN84_05275 JQN84_05335	*M. humidisoli* MMS20-R2-29^T^ *M. humidisoli* MMS20-R2-29^T^ *M. humidisoli* MMS20-R2-29^T^ *M. humidisoli* MMS20-R2-29^T^ *M. humidisoli* MMS20-R2-29^T^
*t1pks-6*	7_29310	99/100	JQN84_14785	*M. humidisoli* MMS20-R2-29^T^
*t2pks-1*	7_20160 7_20170 7_20190	99/100 99/99 99/100	JQN84_23105 JQN84_23110 J7462_05705	*M. humidisoli* MMS20-R2-29^T^ *M. humidisoli* MMS20-R2-29^T^ *Micromonospora* sp. RL09-050-HVF-A
*t3pks-1* * (*aqq*)	7_59200	100/100	JQN84_06220	*M. humidisoli* MMS20-R2-29^T^
*nrps-1*	7_47220 7_47230 7_47240	99/99 99/99 99/100	JQN84_30545 JQN84_30550 JQN84_30555	*M. humidisoli* MMS20-R2-29^T^ *M. humidisoli* MMS20-R2-29^T^ *M. humidisoli* MMS20-R2-29^T^
*nrps-2*	7_47680	99/99	JQN84_14135	*M. humidisoli* MMS20-R2-29^T^
*nrps-3*	7_56920 7_56930 7_56940 7_56970	99/100 99/99 99/99 99/99	JQN84_29450 JQN84_29445 JQN84_29440 JQN84_29425	*M. humidisoli* MMS20-R2-29^T^ *M. humidisoli* MMS20-R2-29^T^ *M. humidisoli* MMS20-R2-29^T^ *M. humidisoli* MMS20-R2-29^T^
*pks/nrps-1*	7_15480	99/99	JQN84_27845	*M. humidisoli* MMS20-R2-29^T^
*pks/nrps-2* *	7_28040 7_28030 7_28010 7_28000 7_27970 7_27960	99/99 99/99 99/99 99/99 98/98 99/99	JQN84_25460 JQN84_25465 JQN84_25475 JQN84_25480 JQN84_25495 JQN84_25500	*M. humidisoli* MMS20-R2-29^T^ *M. humidisoli* MMS20-R2-29^T^ *M. humidisoli* MMS20-R2-29^T^ *M. humidisoli* MMS20-R2-29^T^ *M. humidisoli* MMS20-R2-29^T^ *M. humidisoli* MMS20-R2-29^T^
*pks/nrps-3*	7_56660 7_56670 7_56710 7_56840	99/99 99/99 100/100 99/99	JQN84_29575 JQN84_29570 JQN84_29550 JQN84_29485	*M. humidisoli* MMS20-R2-29^T^ *M. humidisoli* MMS20-R2-29^T^ *M. humidisoli* MMS20-R2-29^T^ *M. humidisoli* MMS20-R2-29^T^
*t1pks-7*	8_40860 8_40870 8_40880	59/69 56/67 54/66	C8E87_8689 M4V62_39485 SBI_01382	*Actinoplanes brasiliensis* DSM 43805^T^ *Streptomyces durmitorensis* MS405 “*Streptomyces bingchenggensis*” BCW-1
*t1pks-8* (*qmn*)	8_45370 8_45410 8_45420 8_45440 8_45450 8_45460 8_45470 8_45480 8_45490 8_45500 8_45510 8_45520 8_45530	98/98 96/96 95/96 91/93 91/93 97/98 98/98 97/97 94/95 97/98 99/99 96/96 96/97	C8054_25705 C8054_25725 C8054_25730 H1D33_RS20350 H1D33_20360 C8054_27580 C8054_27585 C8054_27590 H1D33_20380 C8054_11295 C8054_11300 C8054_11305 C8054_11310	*Micromonospora* sp. RP3T *Micromonospora* sp. RP3T *Micromonospora* sp. RP3T *M. ferruginea* 28ISP2-46 *M. ferruginea* 28ISP2-46 *Micromonospora* sp. RP3T *Micromonospora* sp. RP3T *Micromonospora* sp. RP3T *M. ferruginea* 28ISP2-46 *Micromonospora* sp. RP3T *Micromonospora* sp. RP3T *Micromonospora* sp. RP3T *Micromonospora* sp. RP3T
*t2pks-2*	8_49930 8_49910 8_49900	98/99 99/99 99/99	C8054_23750 CO540_02355 C8054_23735	*Micromonospora* sp. RP3T *Micromonospora* sp. WMMA2032 *Micromonospora* sp. RP3T
*t3pks-1* * (*aqq*)	8_06420	99/98	C8054_27190	*Micromonospora* sp. RP3T
*nrps-4*	8_34180 8_34160 8_34150 8_34130 8_34100	55/66 63/73 55/65 53/66 51/64	ADL15_RS07780 *bnvE* (QVQ62850) HUV60_15065 Raf01_61150 HUV60_15130	“*Actinoplanes awajinensis* subsp. *mycoplanecinus*” NRRL B-16712 *Streptomyces* sp. UTZ13 *Streptomyces* sp. KMM 9044 *Rugosimonospora africana* NBRC 104875^T^ *Streptomyces* sp. KMM 9044
*nrps-5*	8_34870 ^C^ 8_34920 8_35060 8_35080	42/58 44/57 55/67 54/68	KA716_28265 HRW08_08145 SAMN05216553 _119106 DMC61_21850	*Gloeotrichia echinulata* DEX184 *Streptomyces lunaelactis* MM15 *Lentzea fradiae* CGMCC 4.3506^T^ *Amycolatopsis* sp. WAC 04169
*pks/nrps-2* *	8_42740 8_42750 8_42770 8_42780 8_42810 8_42820	99/99 97/97 98/98 99/99 96/96 99/99	C8054_04550 C8054_04555 C8054_04565 C8054_04570 C8054_04585 C8054_04590	*Micromonospora* sp. RP3T *Micromonospora* sp. RP3T *Micromonospora* sp. RP3T *Micromonospora* sp. RP3T *Micromonospora* sp. RP3T *Micromonospora* sp. RP3T
*pks/nrps-4*	8_08330 8_08340 8_08370	87/88 86/88 87/90	GA0070213 _12115 CO540_09565 CO540_09580	*M. humi* DSM 45647^T^ *Micromonospora* sp. WMMA2032 *Micromonospora* sp. WMMA2032
*pks/nrps-5*	8_34600 8_34620 8_34650 8_34660 8_34670 8_34690 8_34690 8_34730	98/98 98/98 96/96 95/96 97/98 97/97 97/97 99/98	C8054_08855 C8054_08865 C8054_08880 C8054_08885 C8054_08890 C8054_08900 C8054_08905 C8054_08920	*Micromonospora* sp. RP3T *Micromonospora* sp. RP3T *Micromonospora* sp. RP3T *Micromonospora* sp. RP3T *Micromonospora* sp. RP3T *Micromonospora* sp. RP3T *Micromonospora* sp. RP3T *Micromonospora* sp. RP3T
*pks/nrps-6*	8_35130 8_35200 8_35210 8_35230 8_35250	52/59 59/69 55/70 64/73 56/68	GCM10011578 _091720 MXD61_11230 LX86_002128 SAMN05216215 _102899 SAMN05216215 _102897	*Streptomyces fuscichromogenes* CGMCC 4.7110^T^ *Frankia* sp. AgPm24 *Lentzea aerocolonigenes* DSM 40034^T^ *Saccharopolyspora shandongensis* CGMCC 4.3530^T^ *S. shandongensis* CGMCC 4.3530^T^
*pks/nrps-7*	8_54560 8_54550 8_54470 8_54430 8_54260 8_54200 8_54120 8_54020 8_54000 8_53990 8_53970	63/76 71/82 57/69 89/94 94/95 89/92 93/95 98/98 96/97 98/98 98/98	Psuf_070260 Psuf_070270 FHG89_16340 DER29_6205 C8054_02625 DLJ59_18505 C8054_02645 C8054_02695 C8054_02705 C8054_02710 C8054_02715	*Phytohabitans suffuscus* NBRC 105367^T^ *P. suffuscus* NBRC 105367^T^ *M. orduensis* S2509 *Micromonospora* sp. M71_S20 *Micromonospora* sp. RP3T *M. inaquosa* LB39^T^ *Micromonospora* sp. RP3T *Micromonospora* sp. RP3T *Micromonospora* sp. RP3T *Micromonospora* sp. RP3T *Micromonospora* sp. RP3T

^1^ Similarity/identity in amino acid sequences. ^C^, encoded in the complementary strand; *, conserved between strains AKA109 and AKA38.

## Data Availability

The whole genome shotgun project of *Micromonospora* sp. AKA109 and *Micromonospora* sp. AKA38 have been deposited at GenBank under the accession numbers BNEH00000000 and BNEI00000000, respectively. BioProject accession numbers are PRJDB9818 and PRJDB9819. BioSample accession numbers are SAMD00228008 and SAMD00228009.

## Supplementary Materials

The following supporting information can be downloaded at: https://www.mdpi.com/article/10.3390/life13020542/s1, Table S1: Secondary metabolite-biosynthetic gene clusters, except for PKS and NRPS gene clusters, of *M. humidisoli* AKA109; Table S2: Secondary metabolite-biosynthetic gene clusters, except for PKS and NRPS gene clusters, of *Micromonospora* sp. AKA38.
