# Supplementary material for: Taxonomic Positions and Secondary Metabolite-Biosynthetic Gene Clusters of Akazaoxime- and Levantilide-Producers
Source: Life (Basel). 2023 Feb 15;13(2):542. doi: 10.3390/life13020542 (PMC9967187; doi:10.3390/life13020542)
Supplement: Supplementary file 1 [file life-13-00542-s001.zip › life-2124316-supplementary.docx]

**Supporting Information**

Taxonomic positions and secondary metabolite-biosynthetic gene clusters of akazaoxime- and levantilide-producers

Hisayuki Komaki ^1,^*, Tomohiko Tamura ^1^ and Yasuhiro Igarashi ^2^

^1^ Biological Resource Center, National Institute of Technology and Evaluation (NBRC), Chiba 292-0818, Japan; komaki-hisayuki@nite.go.jp (H.K.), tamura-tomohiko@nite.go.jp (T.T.)

^2^ Biotechnology Research Center and Department of Biotechnology, Toyama Prefectural University, Toyama 939-0398, Japan; yas@pu-toyama.ac.jp (Y.I.)

***** Correspondence: komaki-hisayuki@nite.go.jp

**Table S1.** Secondary metabolite-biosynthetic gene clusters, except for PKS and NRPS gene clusters, of *M. humidisoli* AKA109

| **Region*** | **Type of gene cluster** | **Predicted product** | **By antiSMASH*** | | |
| --- | --- | --- | --- | --- | --- |
|  |  |  | **Most similar known cluster** | | **Similarity** |
| 5.1 | terpene ^a^ | unpredictable | tetrachlorizine | Polyketide | 13 % |
| 5.2 | thioamide | unpredictable | enteromycin | Polyketide+NRP | 8 % |
| 5.8 | lanthipeptide-class-iii ^b^ | SapB | SapB | RiPP:Lanthipeptide | 100 % |
| 5.9 | terpene | unpredictable | n/a | n/a | n/a |
| 5.10 | siderophore ^c^ | desferrioxamine | FW0622 | Other | 50 % |
| 5.14 | hybrid oligosaccharide/terpene ^d^ | unpredictable | lobosamides | Polyketide | 86 % |
| 5.16 | NAGGN ^e^ | NAGGN | n/a | n/a | n/a |
| 5.20 | terpene ^f^ | unpredictable | phosphonoglycans | Saccharide | 3 % |
| 5.21 | indole | unpredictable | fortimicin | Saccharide | 13 % |
| 5.22 | terpene ^g^ | unpredictable | isorenieratene | Terpene | 25 % |
| 5.23 | indole | unpredictable | gausemycins | NRP+Saccharide | 7 % |

* Results of antiSMASH analysis. Different types of gene clusters are often searched as the ‘Most similar known cluster’ in antiSMASH (Komaki *et al*. *Hydrobiology* **2023**, 2, 151-161). ^a–g^ Conserved between *M. humidisoli* AKA109 and *Micromonospora* sp. AKA38. ^e^ NAGGN, *N*-acetylglutaminylglutamine amide.

**Table S2.** Secondary metabolite-biosynthetic gene clusters, except for PKS and NRPS gene clusters, of *Micromonospora* AKA38

| **Region*** | **Type of gene cluster** | **Predicted product** | **By antiSMASH*** | | |
| --- | --- | --- | --- | --- | --- |
|  |  |  | **Most similar known cluster** | | **Similarity** |
| 1.1 | terpene ^g^ | unpredictable | isorenieratene | Terpene | 25 % |
| 3.1 | terpene ^a^ | unpredictable | tetrachlorizine | Polyketide | 13 % |
| 4.1 | terpene | unpredictable | Lymphostins | NRP+Polyketide | 25 % |
| 5.1 | lanthipeptide-class-ii | RiPPs** | n/a | n/a | n/a |
| 5.5 | lanthipeptide-class-iii ^b^ | SapB | SapB | RiPP:Lanthipeptide | 100 % |
| 5.7 | terpene | unpredictable | Nocathiacin | RiPP:Thiopeptide | 4 % |
| 5.9 | siderophore ^c^ | desferrioxamine | FW0622 | Other | 50 % |
| 5.10 | hybrid of oligosaccharide/terpene ^d^ | unpredictable | lobosamides | Polyketide | 13 % |
| 7.1 | terpene ^f^ | unpredictable | phosphonoglycans | Saccharide | 3 % |
| 8.1 | terpene | unpredictable | lymphostins*** | NRP+Polyketide | 33 % |
| 10.1 | NAGGN ^e^ | NAGGN | n/a | n/a | n/a |

* results of antiSMASH analysis; ** derived from AERAGSTARLSTIAIQFGQCGANVQLR, RAARLPQLHISTTLTELNGDC, and PPAGQLGYRSCTLAPH; *** lymphostin/neolymphostinol/lymphostinol/neolymphostin; ^a–g^ conserved between *Micromonospora* sp. AKA38 and *M. humidisoli* AKA109; ^e^ NAGGN, *N*-acetylglutaminylglutamine amide.
